# Does medical education erode medical trainees' ethical attitude and behavior?

**Published:** 2016-11-23

**Authors:** Neda Yavari

**Affiliations:** PhD Candidate in Medical Ethics, Medical Ethics and History of Medicine Research Center, Tehran University of Medical Sciences, Tehran, Iran.

**Keywords:** *Ethical erosion*, *Medical trainees*, *Medical education*

## Abstract

In the last few years, medical education policy makers have expressed concern about changes in the ethical attitude and behavior of medical trainees during the course of their education. They claim that newly graduated physicians (MDs) are entering residency years with inappropriate habits and attitudes earned during their education. This allegation has been supported by numerous research on the changes in the attitude and morality of medical trainees. The aim of this paper was to investigate ethical erosion among medical trainees as a serious universal problem, and to urge the authorities to take urgent preventive and corrective action. A comparison with the course of moral development in ordinary people from Kohlberg’s and Gilligan's points of view reveals that the growth of ethical attitudes and behaviors in medical students is stunted or even degraded in many medical schools. In the end, the article examines the feasibility of teaching ethics in medical schools and the best approach for this purpose. It concludes that there is considerable controversy among ethicists on whether teaching ethical virtues is plausible at all. Virtue-based ethics, principle-based ethics and ethics of care are approaches that have been considered as most applicable in this regard.

## Introduction

The incentive for this research project was provided by a personal encounter from some years ago, when I was a young intern in a prestigious Iranian hospital. After visiting a patient, doing an accurate physical exam and studying her most recent lab results, I proceeded to record my findings in her chart as per my internship duties. The patient was an old obese woman. After a few moments, the ward resident came in and began to study the patient’s chart. While reviewing the chart, he took a degrading look at the patient and used a nickname related to her obesity. I was really mad at the resident and wanted to object to his unethical behavior. However, I found it safer to conceal my anger and save my friendly relationship with the resident at the moment.

I was really disappointed at what I did. Arguing internally, I remembered the same thing had happened a few years ago, when I was a first-year student. Back then, I had voiced my objection to my superior’s unethical behavior, something that I am proud of to this day.

Contrasting these two experiences, I asked myself what had happened to me during those years? Why had I so dramatically lost sensitivity to the ethical aspects of the job? Had it happened to my friends too? And more importantly, what is the main culprit and how could we regain our ethical attitude, that moral perspective that encourages one’s commitment to ethical behavior?

Carole Kleinman defines “ethical erosion” as “a gradual erosion of ethical behavior that occurs in individuals below their level of awareness” (1). Although some subtle differences exist between this and other scholars’ definitions, most share a focus on gradual changes in medical students’ attitudes and behaviors, changes which present themselves through a halt or regression in moral development during the course of training. Some scholars generalize this erosion to moral reasoning as well as ethical sensitivity, commitment and behavior. Furthermore, some scholars believe this erosion does not happen unconsciously. Unlike Kleinman, they claim medical students are both aware of these changes and worried about their character (2). 

Recent studies show that ethical erosion is one of the most challenging bioethical problems throughout the world. In fact, studies from different countries demonstrate the universality of the problem, which is part of its significance (2-4).

Moreover, due to the deep effect of students’ moral attitudes and behaviors on their characters as future practitioners, ethical erosion among medical trainees is worthy of evaluation. Ethical breaches are intrinsically detrimental, and may deeply and sometimes even permanently affect students’ personality and their attitude toward patients, colleagues, and the profession. 

From the social point of view, loyalty to ethical principles directly aids physicians in practicing medicine. Good clinical practice cannot be separated from professional and ethical behavior that is the result of moral maturity. Therefore, any flaws in the ethical attitudes and behaviors of today’s students will directly affect their clinical performance as future practitioners and seriously challenge the health care system.

Jason Liebowits elegantly demonstrates the importance of medical students’ morality (5). Referring to German physicians’ extensive participation in the Holocaust, he attempts to examine the circumstances that turned physicians from healers into accomplices of genocide in this terrible event. He persists in questioning these doctors’ training, looking for factors that turned them into the agents of genocide. The most important questions are probably his last when he asks if medical education potentially paves the way for moral erosion, and if physicians still are vulnerable to losing their ethical principles. He concludes by wondering what can be done to prevent this regression (5).

In the same article Liebowitz shows the personal and social consequences of disregarding ethical values. He refers to Nazi doctors’ cruel behavior toward prisoners and claims that these tragedies happened because of the physicians’ disloyalty to ethical principles. The self-evident principles were gradually but so extensively ignored that, after a while, unethical behaviors turned from unacceptable deeds into norms. The misfortune started when some doctors lost their safeguards against harming patients and justified progressively greater breaches of professional conduct. As a cumulative result of such misbehavior, the medical community reached a point where there was no turning back: they had already transformed from healers to murderers. According to Liebowitz, German physicians’ participation in the Holocaust demonstrates that inattention to changes in the ethical attitudes and conduct of medical students may result in human tragedies at not only a personal but also a social level (5). 

Another reason for the importance of discussions about ethical erosion among medical trainees lies in the possibility of resolving the problem and preventing its negative consequences through taking corrective action. 

The problem can be examined from several aspects. In this research, findings of studies from several countries will be presented to demonstrate that ethical erosion is a universal and serious problem among medical trainees. Furthermore, the expected course of moral development in ordinary people from Kohlberg’s and Gilligan’s points of view will be introduced and compared with the moral development of medical students. Subsequently, some important ethical approaches will be presented and the advantages and disadvantages of their application to the ethical education of medical students will be discussed. The present study documents the ethical changes in medical students during the course of their education, and can therefore be used to enhance their moral development through preventive and therapeutic intervention programs.

## Materials and Methods

For the purpose of investigating the changes in medical trainees' ethical attitude and behavior during the course of education, several websites including Pub Med, Ovid and Elsevier were examined. In order to validate the results, various resources and published research on the topic were studied as well. The keywords used for this aim were “medical education”, “ethical erosion” and “attitude change”. The hypothesis was that the growth of students’ ethical attitude and behavior is stunted, or even worse, degraded in medical schools throughout the world. 

## Results

**The scope of the problem in medical schools of different countries**

Recent studies point to ethical erosion as a serious problem in medical education in countries such as the United States, the United Kingdom, Canada and Scotland, while the author has seen it first-hand in the Middle East as well. This article will examine recently published evidence of medical students’ moral status to show the universality and seriousness of the problem. 

In a 1989 study by Philip Hébert at the University of Toronto, five clinical vignettes with seven to nine ethical issues each were developed. A randomly selected vignette was given to a first-, second- or third-year student who was asked to report the number of ethical issues in the vignette. Each response was scored based on the number of ethical issues identified by the student, regardless of the content of the response. A statistical analysis of 281 responses showed that the second-year class identified the highest average number of issues, which was 3.13. The average numbers were 2.35 for first-year and 2.46 for third-year students. The increased number of identified ethical issues from first year to second year can be attributed to the fact that all of the students participating in this study passed a course on ethics in their first year of medical education (3).

One year later, in 1990, Hebert and his colleagues conducted a similar study with students in their fourth year. As they had expected, the findings showed a further decrease in ethical sensitivity. In other words, the percentage of issues identified by the subjects increased between the 1st and 2nd year and then decreased throughout the next two years, so that the 4th-year class identified the least number of issues in these vignettes after passing clinical training. It is notable that though the vignettes were slightly different in the two studies, the percentages are remarkably similar (6).

These two studies suggest that as medical students progress in the course of their education, they are more likely to lose their sensitivity to ethical problems. In other words, their ability to recognize bioethical dilemmas – as the first step in a logical encounter with these problems – decreases gradually. This regression is considerable particularly after clinical training, and urges us to think about some aspects of clinical training as a probable root for the adverse effect on students’ ethical attitudes and behaviors.

A 1992-1993 study by Chris Feudtner et al. involving 1853 third- and fourth-year medical students in six Pennsylvania medical schools showed that students were aware of and concerned about erosion of their ethical attitudes and behaviors. This study revealed that 67% of the subjects felt guilty about their behaviors, and 62% believed that at least some of their ethical principles had been lessened or lost. Furthermore, 58% of the respondents had done something they believed was unethical, 52% had misled a patient during their practice, and 80% reported at least one of the two (2).

In 2003 Patenaude et al. published the results of studies conducted in Canada that suggested a negative trend in students’ ethical growth during medical training. These studies demonstrated either decreased or inhibited development in the moral attitude and behavior of medical trainees (7).

In one of these studies, students in Quebec were invited to complete a questionnaire on moral reasoning once before their first year of medical school and once after their third. Of the 92 entering medical students, 54 completed the questionnaire twice. The Moral Judgment Interview (MJI) scale (developed by Lawrence Kohlberg in 1958) was used to measure the changes in students’ moral reasoning. Kohlberg believed that people gradually proceed through sequential stages of moral development, even though the rate of progress and the final stage they reach vary by individuals. After students’ responses were collected, they were coded according to parameters described by Colby and Kohlberg, and the answers for each question in the questionnaire were ascribed a score varying between 2 and 5. Subsequently, the most frequent score was assigned to each respondent as his/her moral development stage, and if two scores had exactly the same frequency, an intermediate score was given. 

Thus, two scores for moral development were attributed to each student for the first and third year of medical school. The students' stages of moral development in their first and third years of medical school were compared and they were divided into 3 groups: 

*Group 1* **:** A total of 39 students (72%), who did not show any substantial changes in the stage of moral reasoning from year 1 to year 3

*Group 2* **:** A total of 8 students (15%), who showed progress in the stage of moral reasoning after 3 years

*Group 3* **:** A total of 7 students (13%), who showed decreases in the stage of moral reasoning (7).

As the next step, two weighted average scores were defined for the first- and third-year questionnaires by calculating the average of all scores attributed to different questions in each questionnaire. 

The conclusion was that the differences in average changes in moral reasoning were not statistically significant. However, the mean changes in weighted average scores significantly declined. In fact, all seven students who had experienced a decrease in stages of moral development, and the 39 students who had undergone no change, showed significant decreases in weighted average scores. Therefore, decreases in the weighted average score happened to 45 of 54 students. Patenaude et al argue that, similar to some studies in the United States, they did not observe any progress in the development of moral reasoning among Canadian students as might be expected. They concludes that the society needs some urgent action to provide students with an educational curriculum that enables them to keep their stage of moral development, if not increase it by medical education (7).

Recent studies show that a large number of medical students cheat on exams in medical schools (8, 9). In a study in Scotland, Roff and Preece found that about a quarter of medical students had written ‘‘nervous system examination normal’’ in some patients’ notes even when they had not carried out the examinations (4). Also, a significant number of residents in a U.S. training program had falsified their credentials when applying for fellowships (10). Daniel Sulmasy claims that a surprising number of students not only do not maintain their moral virtues, but also adopt some unethical behaviors in order to survive stressful clinical situations (10). 

The above-mentioned studies show the prevalence and importance of the problem of ethical erosion among medical trainees in several countries around the world. 

**The course of moral development**

Lawrence Kohlberg and Carol Gilligan are two psychologists who have worked on young adults’ moral development. Kohlberg classifies three general levels of moral development: pre-conventional, conventional, and post-conventional (11). He explains that for younger children – who are considered in the pre-conventional stage – “right” is what keeps them from punishment, and they are not able to generalize their findings to other situations. In the conventional stage, that is, later childhood and adolescence, individuals adjust their behaviors based on others’ approval and to exhibit loyalty. They try to fit the society by adopting behaviors that are in line with social norms. At this stage, people have a sense of self and are able to generalize their ethical findings to similar situations. In higher sub-stages of the conventional level, “individuals learn a more abstract understanding of their own roles, obligations, and customs as well as others” (11).

The last stage, or the post-conventional level, is characterized by governing one’s behavior through moral principles. Kohlberg describes an even higher sub-stage at the post conventional level, one that is achievable for only a limited number of individuals, when “the principles of human dignity and human rights are integrated into the personality and form the basis of a person’s action” (11).

**Medical students and Kohlberg’s concept of ‘moral maturity’**

Students are not all at the same developmental level, and therefore respond differently to the ethical challenges in clinical environments. Medical students usually enter the university between the ages of 21 and 24, which is the age when individuals are expected to begin their passage to the post-conventional level according to Kohlberg’s classification. However, available data suggest that this progress is not fully accomplished in many students, and ethical dilemmas challenge their principles in many cases. As a result, some accept the conventional moral guidelines of the surrounding environment, whereas others express their discomfort at being assimilated into the ethical and social norms and the demands of the clinical climate, and strive to maintain their moral values. The latter group continues its moral development in spite of all adversity. Nevertheless, in their descriptions of this conflict, students express concern about possible regression to the lower moral levels (12). 

As Kohlberg suggests and other studies confirm, medical students react to this conflict differently. While many students struggle to accommodate their personal values within the scope of clinical training, others may be pressured to conform to the wards’ culture and give up those personal ethical values that are in contrast with their ability for rapid decision-making. However, the moral maturity of the latter is generally recognized as paused or even degraded (12-14).

Kohlberg describes the transition from the conventional to the post-conventional stage as the development of “moral reasoning”, which he claims to be only one component of ethical behavior. Ethical behavior is in fact a combination of moral sensitivity (the ability to recognize ethical problems), moral commitment (a strong intention to do the right thing), moral behavior (skills at implementation), and moral reasoning (the ability to balance others’ rights against the principles at stake (12).

In other words, ethical behavior is a combination of moral reasoning, moral sensitivity, moral commitment and moral behavior. The importance of moral reasoning should not impede our attention to those other aspects of ethical behavior. Students are confronted in wards with demands for action, and supporting all components of ethical behavior is therefore essential for their moral development (12).

Carol Gilligan worked on Kohlberg’s theory later and, unlike him, emphasized “connection, care and response” in moral development. She believes that these factors complete the Kohlberg approach by focusing on “equality, justice, and rights”, because emphasizing mere justice as the index of morality results in impartiality and consequent indifference. Thus, Gilligan argues that caring and justice are both moral focal points and should therefore be promoted as complementary factors (15).

Branch et al. observed that students are more concerned about hindrances that pose a threat to their compassion and care rather than their reasoning abilities in confrontation with ethical issues. They usually place a high value on openness, sensitivity, and an understanding of patients and their problems. One can say that students generally pay more attention to the caring aspect of their job than their role as members of the clinical team. Branch et al. state that caring, as opposed to moral reasoning, continues to remain the most important ethical issue for the typical medical student. Thus, promotion of moral reasoning should not be the only goal in bioethical education, and medical students should be equipped with an integrated attitude toward morality (12, 16). 

**Is teaching ethics plausible at all?**

Prior to discussing the most effective method for ethical training in medical schools, one needs to answer whether teaching ethics to medical trainees is plausible at all.

Edmund Pellegrino believes that the teachability of virtues has been questioned since Plato and Socrates. He points out that Aristotle's works have suggested a significant method for the development of students’ professional character: providing them with virtuous role models. Aristotle states that the best practice for learning virtues is to follow the example of a virtuous person. In medicine, therefore, we need virtuous role models among physicians, as they are highly responsible for the characteristic traits that they exemplify for trainees. Pellegrino asserts that even though medical students’ characters have been shaped to a certain extent prior to entering medical schools, they continue to remain impressionable even as they start their careers as physicians (17). 

Thus he argues that “courses in medical ethics, the humanities, human values, etc., can sensitize, raise awareness and force critical reflection about the virtues of the good physician. [Ethics] courses introduce students to a body of literature which gives evidence of the importance, depth and complexity of the moral issues commonplace in medical practice” (12).

On the other hand, Daniel Sulmasy speculates whether ethics courses can help produce morally better doctors. Simply put, he examines the teachability of virtues. Contrary to those who argue that students enter medical schools “already morally packaged and incapable of change” (10), Sulmasy believes that ethics courses can indeed be effective, and that it is essential to create a nurturing academic environment in which professional virtues receive real attention. It is possible, he states, to “consciencitize” students and provide them with the knowledge and skills necessary to deal with medical ethics issues. However, Sulmasy argues, that these courses cannot change the individuals’ attitudes. As an example, he mentions a physician who has been educated on the principles of obtaining informed consent, but behaves in a disparaging manner that will not be reflected in a consent form. He concludes that teaching virtues can be considered only as a complement to ethical theories, not as a substitute for them (10).

Unlike Sulmasy, William Branch examines the data and concludes that changes in students’ attitudes are possible, but may happen in the wrong direction. These studies demonstrate that virtues such as compassion and altruism are unfortunately undermined due to the current trends in medical education (10).

## Discussion

Many philosophers of ethics agree on the necessity of specialized courses in medical schools in order to minimize ethical erosion in trainees. The next step would be to select from different ethical approaches the best to be taught via the educational curricula in medical schools. In other words, the question is which theories will maximize medical students’ chances of adopting ethical behaviors in complicated situations. Virtue-based ethics, principle-based ethics and ethics of care are approaches that have been considered as most applicable in this regard.

Virtue ethics, which can be followed in Western thought to the works of Plato and Aristotle, emphasizes the character of the agent (18). Sulmasy defines virtue as “the critical aspect of ethics that deals with character” (10), and believes this concept should guide medical students become the kinds of doctors they ought to be. He describes medical virtue as all characteristics of a good doctor including scientific competence, compassion, wisdom, courage and patience. A doctor committed to these virtues, in his view, is a trustworthy doctor even when left unsupervised (10).

Proponents of virtue-based ethics contend that medical students must be trained to perform moral acts because they possess certain virtues such as benevolence or generosity. The next step for the advocates of this approach is to determine which virtues are desirable and how they can be developed (18).

Scholars have emphasized the effect of virtues in providing the physician with a humane perspective on patients. Pellegrino, for instance, claims that the virtue-based doctor is almost incapable of viewing his patient as a “customer, consumer, insured life or any other commercialized, industrialized transformations of the ancient and respectable word ‘patient’. Nor could he compromise his personal or professional integrity for political, economic, or social advancement. Nor could the virtuous physician become a union member, go on strike, or engage in blatant self-promotion and advertising even though it is sanctioned by law. This would suit the ethics of professionalization but not of a true profession” (17).

In a different approach, the deontologist is more concerned about the action to be performed in any given situation (18). Lynn Jansen believes that virtue ethics promotes medical ethics and can play a significant role in improving students’ moral development. However, when students face a moral conflict or dilemma, this approach does not support them in identifying morally correct actions or behaviors. It only shows whether the action possesses any moral worth or not. In order to adopt the right behavior, students depend on other concepts such as rules, duties and principles. Jansen asserts that teaching virtue-based ethics to medical students poses two main problems. First, it is sorely difficult to determine a person’s motivation for taking an action. Second, while the ultimate goal of medical education should be to preserve the interests of patients and their families, this approach places more value on the right motivation rather than a patient-centered practice. Jansen declares that a good character is important as a means to good conduct, but due to the above-mentioned problems, it is a mistake to apply virtue ethics to medical practice (19).

Jansen claims that a worthy action has two components: decent motivation and rightness of behavior. The genuine role of virtue-based ethics is to teach medical students to do right things for good reasons. “By training virtuous characters in medical students we prepare them to do morally worthy actions in hard and complicated medical situations, not simply actions that are in line with what they ought to do” (19).

Principle-based ethics supports the rules or ethical principles that one may follow. This approach does not take into account the personality or intention of individuals. Therefore, the right action is defined as the one conducted based on accepted principles (18). Bioethicists have made several criticisms of principle-based theory. Tom Beauchamp and James Childress, for example, believe that a pluralistic principle-based theory works best because it emphasizes a number of principles of obligation. In their opinion, single principle methods such as utilitarian ethics (the ethical theory with the aim of providing the most good for the most people) and Kantian ethics (a single principle-based deontological model) may not be as effectual. Unlike other theories that define a supreme or absolute principle that supports all other action guides in the system, the pluralistic approach offers several non-absolute (prima facie) principles of equal importance, and entrusts ethical decision-making to the person applying these principles. Furthermore, in contrast to utilitarian and Kantian theories that rely on pure reason, the pluralistic principle-based theory relies on common sense morality. The principles in turn work as indicators for moral judgment in any particular case. Any customary morality is accepted in this system only if it is in good balance with the other rules and principles of the theory. New attitudes and practices are evaluated in terms of compatibility with this theory through John Rawls’s process of reflective equilibrium and, if coherent, are considered morally acceptable (20).

Beauchamp and Childress’s principle-based theory defines four principles in the realm of medical ethics: autonomy, beneficence, non-maleficence and justice. Critics of this theory assert that these principles are too general and ambiguous to be used as specific action guides. Therefore, medical students may experience ambiguity and uncertainty when applying these ethical standards in particular cases. Moreover, opponents of this model argue that the reliance of these principles on commonsense morality rather than pure reason, natural law or a special moral sense discredits the principles by creating a kind of ethical relativism. K. Danner Clouser and Bernard Gert believe that since each principle has its own logic and life, a number of internal conflicts leading to a kind of relativism are inevitable. They and other critics claim that defined principles in medical ethics at best work as a checklist of points to consider in the face of moral problems. By this critique they deny the value of guiding particular action by applying the pluralistic principle-based theory (20). Considering these possible flaws in the pluralistic ethical model, it seems wise not to rely upon it as the only ethical theory to be taught in medical schools.

The ethics of care is the next theory that has been widely considered in the moral training of medical students. This theory was derived from Gilligan as a response to principle-based ethical theories. Contrary to proponents of principle-based theories, Gilligan dismisses impartiality or disregard for others’ values in moral reasoning and recommends moral judgment based on situation-attuned insight into each case instead. She encourages sensitivity to others’ needs and applying norms of responsiveness and responsibility in relationships. Alisa Carse, one of the pioneer advocates of care ethics in medicine, emphasizes the role of emotions in moral discernment and moral reasoning, and stresses that a timely and appropriate communication of the right emotions is a basic characteristic of moral agency (15). Acknowledgement of emotions in ethical decision-making has turned care ethics into one of the most popular ethical theories for moral training of medical students. After all, it seems that emotional attunement and sympathetic insight are essential to establishing a humane relationship between physicians and patients.

## Conclusion

The studies described here show the prevalence and importance of ethical erosion among medical trainees in several countries around the world. Documenting the ethical changes in medical students during the course of their education, this work can be used to guide the moral training of medical students through preventive and therapeutic intervention programs.

Kohlberg and Gilligan define three levels of moral development and believe that usually people develop toward higher levels as they get older. Although medical students are often at an age when according to Kohlberg and Gilligan should progress toward the post-conventional level, available data suggest that this progress is not completed in many students. The challenging nature of ethical dilemmas forces a notable number of students to accept the conventional guidelines of the clinical environment and keeps them from developing their morality toward the expected higher levels. 

Furthermore, there is considerable controversy among ethicists over the plausibility of teaching ethical virtues. William Branch believes that in a nurturing environment where professional virtues receive real attention, medical students are able to develop their moral attitude and behavior. 

He contends that medical students would be able to transition to higher levels of moral development if they were exposed to a morally acceptable educational environment. Virtue-based ethics, principle-based ethics and ethics of care are approaches that have been considered as most applicable in this regard.

Future studies might compare the ethical attitude and behavior of physicians to that of other service-oriented professions such as lawyers or university professors. Such a comparison might shed a more comprehensive light on effective factors in professional education and the corrective practices medicine might adopt. 

Moreover, the studies discussed in this article were limited to medical students, and it is not clear if ethical erosion continues in working physicians or somehow stops when the new doctors take more responsibility as they start practicing medicine. Therefore, one interesting subject for further study could investigate the possible changes in ethical attitude and behavior after residency.
